# Continuous deep sedation and the doctrine of double effect: Do physicians not intend to make the patient unconscious until death if they gradually increase the sedatives?

**DOI:** 10.1111/bioe.12792

**Published:** 2020-07-14

**Authors:** Hitoshi Arima

**Keywords:** continuous deep sedation, doctrine of double effect, gradual CDS, intention, permanent unconsciousness, proportionate palliative sedation

## Abstract

Continuous deep sedation (CDS) has the effect of making the patient unconscious until death, and that it has this effect is clearly an undesirable aspect of CDS. However, some authors have recently maintained that many physicians do not intend this effect when practicing CDS. According to these authors, CDS is differentiated into two types; in what is called “gradual” CDS (or CDS as a result of proportionate palliative sedation), physicians start with low doses of sedatives and increase them only gradually, whereas in “rapid” CDS (or palliative sedation to unconsciousness), physicians rapidly administer a heavy dose that clearly induces unconsciousness from the beginning. The claim is that the physicians intend permanent unconsciousness only if they rapidly administer a heavy dose, but they do not intend it when the unconsciousness is the result of a gradual increase of sedatives. This paper attempts to refute these claims based on a close examination of the protocol of gradual CDS. If my argument is valid, the doctrine of double effect would not be useful in justifying most, if not all, cases of CDS.

## 1 INTRODUCTION

It is often believed that there is no need for euthanasia because most types of suffering can be effectively alleviated by measures of palliative medicine and, in fact, any suffering can be removed if continuous deep sedation (CDS) is employed. Simultaneously, however, many clinicians and researchers also doubt if CDS is any better than euthanasia ethically.

The doubt is based on two facts about the nature of CDS: one is that CDS potentially shortens the patient’s life, and the other is that it renders the patient unconscious until death. A literature review shows that discussions on the morality of CDS have mostly focused on the first fact, and almost completely ignored the latter until recently.^1^ Arguably, however, it is the latter fact that makes it more difficult to distinguish CDS from euthanasia ethically. For although it is at least debatable if CDS can hasten death when properly conducted,^2^ there is no room to doubt that it renders patients permanently unconscious (that it does so is part of the definition of CDS). Also, there is good reason to believe that being rendered permanently unconscious is just as bad as being killed.

Sedation, as it is used in palliative care, is classified as either continuous or intermittent, and as either deep or light. Sedation is called continuous if it is continued until the patients’ death, and intermittent (or respite) if it is not. Deep sedation refers to cases where the patients are rendered unconscious, whereas in the light cases patients do not lose consciousness completely.^3^ Thus, CDS is a palliative measure that, by definition, renders patients unconscious until death.

This is clearly an undesirable character of CDS. While euthanasia brings about death to patients, CDS brings about a life without consciousness to them. At least from the patients’ perspective, a life without consciousness has no more value than death, since these two states are subjectively indistinguishable.^4^ Hence, if euthanasia is morally wrong (as is often believed), and its wrongness is to be explained solely by its impact on the patients’ experience, CDS seems to be just as wrong as euthanasia.

Now, the defenders of the belief that CDS is morally better than euthanasia commonly appeal to difference in the physicians’ intentions. If CDS and euthanasia are equally bad in their consequences, it is argued, palliative physicians do not intend to bring about the bad consequence, while physicians carrying out euthanasia do and, therefore, CDS is less objectionable than euthanasia. In the background to this argument lies the doctrine of double effect (DDE), a moral rule that justifies one’s knowingly bringing about a bad consequence, on the condition that, among other things, the agent only foresees, but does not intend, the bad consequence.^5^

Again, most discussions regarding the validity of this line of argument have so far focused on CDS’s life‐shortening effect as the relevant bad consequence.^6^ More recently, however, the consciousness‐removing effect of CDS has gained some serious attention,^7^ and the question of whether physicians who practice CDS intend permanent unconsciousness has also become a target of investigations. While some researchers do not doubt that physicians who practice CDS intend permanent unconsciousness,^8^ some claim otherwise.

Timothy Quill and colleagues maintain that CDS is further differentiated into two types, depending on the way physicians administer sedatives.^9^ Some physicians start with low doses of sedatives and increase them only gradually, while others rapidly administer a heavy dose that clearly induces unconsciousness right from the beginning. According to Quill and colleagues, physicians intend permanent unconsciousness only when they rapidly administer a heavy dose, but they do not intend it when the unconsciousness is the result of a gradual increase of sedatives. This suggests that many physicians who practice CDS do not in fact intend permanent unconsciousness, or that they can avoid forming such intention. By now, these claims of Quill and colleagues are accepted by a number of other researchers writing on this subject.^10^ The purpose of this paper is to examine these claims and show that they are mistaken.

Section 2 of this paper introduces the distinction between gradual CDS and rapid CDS in more detail to prepare my criticism. Then in section 3, I criticize the claim that physicians do not intend permanent unconsciousness in cases of gradual CDS. My criticism borrows from an analysis on the nature of human intention developed by Daniel P. Sulmasy. Sulmasy also discusses the morality of palliative sedation and draws conclusions that are somewhat different than mine. In section 4, I point to the merits of my argument over Sulmasy’s. On these bases it will be concluded that physicians must be understood to intend permanent unconsciousness when they practice CDS in most, if not all, cases and that, therefore, if euthanasia is deemed impermissible, then most cases of CDS should also be considered impermissible for consistency’s sake. However, the ensuing discussion also shows that there is room for debate regarding whether physicians can practice CDS without forming this intention on rare occasions, and how rare such occasions are is a matter for future empirical investigation.

## 2 THE DISTINCTION BETWEEN GRADUAL AND RAPID CDS

It is maintained that CDS is further differentiated into so‐called gradual and rapid CDSs. The distinction has been explained either by the difference in protocols alone, or by this difference together with the difference in physicians’ intentions. Since I shall eventually argue against the claim that physicians’ intentions differ depending on the type, here I first describe the distinction in terms of the difference in protocols, and then explain how it is understood to entail the difference in intentions.^11^

The difference is said to lie, in the first place, in the amount of sedatives that the physicians begin with and the pace of increasing it. In gradual CDS, physicians begin with the minimum amount of sedatives that they believe is necessary to alleviate the patient’s suffering, but it turns out that the dose is not enough; then the medication is gradually increased at a pace that is considered proportionate to the severity of suffering, with the result that the patient needs to be kept unconscious until death. An international interview research suggests that this is a more common route to reach CDS among palliative care specialists working in the United Kingdom.^12^ In contrast, in rapid CDS, physicians administer sedatives in large amounts that clearly induce unconsciousness right from the start, or rapidly increase the sedatives within a short period of time (within minutes or a few hours) until unconsciousness is induced, and keep the patient in that state until the patient dies. The same interview research shows that this practice is more common in Belgium and the Netherlands.^13^

The distinction is important for our discussion because it is understood to entail a difference in physicians’ intentions. Quill and colleagues maintain that when CDS is a result of a gradual increase of sedatives, “the end point is relief of suffering,” and induction of unconsciousness is “considered a foreseen but unintended side effect when lesser degrees of sedation were ineffective.” However, in other cases, “sedation is rapidly increased over minutes or a few hours until the patient is unresponsive,” and here “unconsciousness is the intended goal of the sedation rather than a side effect.”^14^ As Quill and colleagues understand, the latter practice is ethically controversial, and whether to provide it to patients should be left to the conscientious choice of individual physicians.^15^

Thus, according to Quill and colleagues, physicians who practice CDS do not necessarily intend to render patients unconscious until death. When CDS is gradual, it is understood that they do not have this intention. The distinction is accepted by a number of other researchers including medical professionals and ethicists, and these researchers generally follow the understanding that it entails this difference in physicians’ intentions.^16^ Based on this understanding, it may then be argued that in cases of gradual CDS, physicians are less culpable than those practicing euthanasia, even though CDS renders patients permanently unconscious and being rendered permanently unconscious is just as bad as being killed.

How are we to evaluate this defense of CDS? In the next section I shall attempt to show an important flaw in the defense, but before doing so, let me provide a note on terminology, and some comments on the scope of the defense.

First, a note on terminology. When Quill and colleagues first suggested a distinction between two types of palliative practices, they called them proportionate palliative sedation (PPS) and palliative sedation to unconsciousness (PSU). Later, based on this suggestion, Morita and colleagues proposed that CDS can be differentiated into gradual and rapid. Importantly, while PSU and rapid CDS can be understood as synonymous, PPS and gradual CDS are not. As Quill and colleagues understand, PPS refers to cases where “the pace of increasing the sedation […] rang[es] from hourly to daily”, but it only “[o]ccasionally […] requires sedation to unconsciousness.”^17^ In contrast, Morita and colleagues define gradual CDS as a type of CDS: as they explain, it is “[c]ontinuous deep sedation as a result of proportional sedation.”^18^ If we follow their use of the terms strictly, then PPS is understood as a practice that may or may not result in CDS, and only when it results in CDS is it called gradual CDS. Thus, the former stands for a range of practices that is wider than, and includes, what the latter signifies. Although other writers are not always careful to attend to it,^19^ this difference will be crucial in this discussion.

Lastly, a comment on the scope of the above defense of CDS. It may be thought that the defense has an apparent limitation, namely that it only purports to establish a moral difference between gradual CDS and euthanasia. Obviously, it has no use in defending rapid CDS, for it is not intended to show that rapid CDS and euthanasia are morally different in terms of the physician’s intention.

However, this may not be considered a serious limitation. For one thing, as Quill and colleagues estimate, “[p]roportionate palliative sedation may be relatively common, whereas PSU should be quite rare.”^20^ If this is true, the above defense would suffice to reckon most, if not all, physicians who practice CDS innocent. Further, some may believe that rapid CDS is morally unacceptable. Thus, Quill and colleagues assert that whether to provide rapid CDS or not should be left to individual physicians’ choice. This would mean that a physician can avoid undertaking what is required to intend permanent unconsciousness. Alternatively, others may believe that no physician should provide it to the patients at all. For those believing this way, there would be no need to defend rapid CDS in the first place. In a commentary to Quill and colleagues, Victor Cellarius and Blair Henry state that the amount of sedatives must always be proportionate to the severity of symptoms. In some cases physicians may use a heavy dose of sedatives that induce unconsciousness, but that is only because the severity of symptoms demands it. Hence, for them, there is no exception to “the ethical imperative that physician’s intention in using sedation is to palliate […].”^21^ Cellarius and Henry may well agree that only gradual CDS requires a defense, and therefore the above argument suffices to defend all that needs to be defended.

## 3 IS PERMANENT UNCONSCIOUSNESS NOT INTENDED IN GRADUAL CDS?

I argue that the above defense of CDS is critically flawed in the following way. Quill and colleagues maintain that while permanent unconsciousness is intended in rapid CDS, it is not intended in gradual CDS (or, using their terminology, not intended when CDS is a result of PPS). I shall show that this is mistaken. Even in gradual CDS, physicians must be understood to intend permanent unconsciousness in most, if not all, cases.

The basis of my argument is the following general understanding about the nature of human intention, elucidated by Sulmasy: when an agent intends to achieve some end, but understands that occurrence of another event is a necessary causal condition for its achievement, then it cannot plausibly be denied that the agent also intends the latter event.^22^ Sulmasy proposes this as one of many conditions he sets forth for the plausibility of statements that do not ascribe a certain intention to an agent. The examples Sulmasy provides include the case of a physician who is applying electroconvulsive therapy (ECT) to a manic patient. If the physician claims to have the intention to treat mania but denies to have the intention to produce electrochemical changes in the patient’s brain, the claim is incoherent and hence implausible, because production of electrochemical changes in the brain is necessary for the treatment of mania in ECT.^23^ Another example is a physician who is performing surgery for tubal ectopic pregnancy. If the physician states that she intends to recover the mother’s health but does not intend to remove the fetus, the statement is also incoherent and implausible, because the removal of the fetus is necessary for the recovery of the mother’s health.^24^

Sulmasy sees no need to argue for the truth of this general condition; he asserts that once its content is understood, it is “self‐evidently true.”^25^ Its truth may appear less evident to others, however. The condition seems plausible with respect to cases where the agent is aware of, and needs to work on, the intermediate steps that are causally required to realize the goal, but that is not always the case. Imagine I give my patient a drug to treat a liver disease. Causally the way the drug works is that it alters the structure of cells in the liver. The alteration of cell structure is thus a necessary causal condition for the goal I am intending. However, it seems at least not clear whether I should be said to be intending it. The problem is more obvious in cases where there are unknown intermediate steps. For years people did not know how aspirin worked. It is difficult to believe that they were nonetheless intending all the intermediate causal steps that led to the relief of headache.^26^ Thus, Sulmasy’s condition is likely to require some qualification regarding its proper range of application. Still, it seems that the condition can be safely applied to cases where the agent needs to work consciously and directly on the intermediate steps to achieve the intended goal.

With these preliminary remarks, I shall now argue that most, if not all, physicians who practice gradual CDS understand it is necessary to keep the patient unconscious until death in order to stop suffering. Importantly, a close look at a typical protocol of gradual CDS should show that the physicians’ understandings do not remain the same from when they begin to administer sedatives until the patient’s death. Thus, it is indeed likely that when they begin to use sedatives, many physicians do not think it necessary to induce unconsciousness or to maintain it until death. However, this is not so after the level of sedation is gradually increased. Accordingly, although the contention that physicians do not intend permanent unconsciousness in gradual CDS is plausible for the earlier stages of the protocol, it is implausible for later stages.

Let us look at the protocol more closely. In the gradual CDS (or CDS as a result of PPS), as described by Quill and colleagues, when physicians begin to administer sedatives, they do not know if that will compromise the patient’s consciousness eventually. They start with a small amount of medication that they are sure will not induce unconsciousness. At this moment, they think it is possible either that the initial amount is enough, or that it needs to be increased only to the level that does not remove consciousness, to stop suffering. Further, even in those cases where, as it turns out, the medication needs to be increased to the level that makes the patient unconscious, that can be temporary; it is still possible that physicians would not know if the patient needs to be kept unconscious until death. In fact, they may reduce medication to see if the patient would still suffer when regaining consciousness, and decide that the patient should be kept awake if the patient no longer experiences unbearable degree of suffering. As with any of these moments, if the physicians deny having the intention to keep the patient unconscious until death, I believe the denial can be plausible.

Two important notes are in order, however. First, even at any of these moments, a physician may intend to induce permanent unconsciousness. In general, there is nothing incoherent in supposing that someone intends the occurrence of an event that may or may not result from one’s own act, as in the cases of, for instance, someone who shoots an enemy from distance, or someone who proposes to the object of one‐sided love. It is plausible that they intend to kill or marry, although they are not sure if they will succeed.

Secondly, and more importantly, the gradual CDS only refers to those cases where sedatives are eventually increased, albeit gradually, to the point that the patient becomes unconscious and the physician maintains this unconscious state until death. (If a smaller amount of sedatives is enough to stop suffering, or the patient regains consciousness before death, the practice is not CDS, be it gradual or rapid.) In these cases, it seems most likely that sometime before the patient’s death, the physicians obtain the understanding that the severity of symptoms requires the patient be kept unconscious until death.

Thus, consider the moment where the amount of sedatives has been increased gradually, and then, as the patient continues to suffer unbearably, it is decided to raise the level of sedation again. The medication may be increased by the minimum amount which could possibly make the patient’s condition better. Nonetheless, this increase induces unconsciousness, and the patient is going to be kept unconscious until death. This is what occurs eventually in gradual CDS. Now, my point is that, in the first place, it is at least possible, and indeed likely, that the physicians are also sure about these prospects when they make the decision. It seems plausible to assume that when it is the case that another increase of medication will induce unconsciousness, physicians understand that it will do so most of the times. Also, when the induced unconsciousness should continue until the patient’s death, it is likely that they understand it as well: this is presumably because the effect of the added dose will clearly last longer than the life expectancy of the patient; or, alternatively, because the physicians are determined to maintain the same level of medication until death since clearly the nature of the symptoms would not allow the patient to regain consciousness without suffering unbearably.

I also contend that, in the second place, when physicians have this understanding, it is not possible for them to avoid intending permanent unconsciousness. For it must be said about them that they both intend to stop suffering and understand that it is necessary to induce permanent unconsciousness in order to stop suffering. Some of them may still deny this intention, but the denial would be incoherent and incredible.

While I believe that these are correct descriptions of the typical cases of gradual CDS, I do not deny other possibilities. It may still seem possible that a physician believes another addition of sedatives may not cause permanent unconsciousness when in fact it does. Important questions are whether such cases are possible, and if they are, how frequently they occur. Although providing solid answers to these questions requires empirical investigations, I surmise that such cases are rather infrequent or rare, if possible.

For example, consider those cases in which the patient is still conscious and has a very short life expectancy, say within hours, when the physician increases the medication by a small amount. Suppose the amount of increase is on the borderline between what should clearly induce unconsciousness and what not. Here it is conceivable that the physician believes that the increase may or may not induce unconsciousness, and, as it turns out, the patient is rendered unconscious and dies before she wakes up again. When this occurs, physicians can plausibly deny the intention to induce permanent unconsciousness, although, to repeat the above point, this does not guarantee the absence of such intention (i.e., people often intend what they may not succeed in bringing about). In contrast, in cases where the patient is kept unconscious for a longer period, say a day or longer, that is presumably because of the physician’s preceding decision to keep the patient so. If the medication is added once or more after the initial inducement of unconsciousness and the unconsciousness is thereby maintained until death in fact, then it is more difficult to imagine that, during these hours or days, the physician retains the thought that the patient may regain consciousness before death.

To put it carefully, my conclusions are twofold. Some researchers claim that while permanent unconsciousness is intended in rapid CDS, it is not intended in gradual CDS. However, firstly, I argued that this claim is mistaken. It is safe to say, at least, that even in gradual CDS, there are situations that do not allow physicians to deny their intention to induce permanent unconsciousness in a plausible manner. Secondly, I argued further that in fact it must be rare that physicians can plausibly deny such intention. While I did explain the basis of this latter estimation, whether this estimation is correct or not is ultimately an empirical question, which has not been investigated properly, to my knowledge, until now.^27^

## 4 A BROADER CONSTRUAL REGARDING THE BAD CONSEQUENCE OF SEDATION

My judgments on the plausibility of descriptions about physicians’ intentions are based on the condition of plausibility proposed in an article by Sulmasy. Now Sulmasy himself does not apply the condition to the case of physicians using sedatives in the same article. However, he discusses the ethics of sedation in another, more recent, article, where he draws conclusions somewhat different than mine. Before concluding this paper, I explain where the differences lie, and point to the merits of my argument over Sulmasy’s.

In the new article, Sulmasy does not discuss the plausibility conditions, at least not explicitly. However, he proposes a number of conditions that need to be satisfied for an act to be justified by DDE. One of these conditions requires that, when an act has both good and bad effects, the bad effect is not intended by the agent, and that “the bad effect is not the means by which the good effect is accomplished.”^28^ Sulmasy then refers to Quill and colleagues’ distinction between PPS and PSU, and considers if PPS is justified by DDE. It is pointed out that in PPS (which Sulmasy renames as “parsimonious direct sedation”) sedatives work to remove suffering by way of causing “the suppression of the awareness of symptoms,” and therefore the suppression of awareness must be understood as a means by which the removal of suffering is accomplished. Simultaneously, Sulmasy contends that the suppression of awareness needs to be considered a bad event, and thereby concludes that PPS is not justified by DDE.^29^

The range of practices Sulmasy deems unjustifiable by DDE is wider than what I argued to be problematic in the preceding section. For Sulmasy, DDE is of no use in justifying the entire practice of PPS, whereas the target of my criticism was restricted to gradual CDS, which is a part of PPS. Behind Sulmasy’s blanket opposition to PPS is a broader understanding of what constitutes the bad effect of sedation. Sulmasy denies seeing any morally relevant difference between reduced consciousness and permanent unconsciousness. For him, aiming “to diminish consciousness” and aiming “to render the patient totally unconscious”^30^ are equally prohibited by DDE as instances of intending a bad effect. (And, for that matter, unintentionally diminishing consciousness as a means to stop suffering, and unintentionally rendering the patient totally unconscious as a means to stop suffering, are equally prohibited by DDE.^31^) Sulmasy in fact uses strong expressions to condemn the former attitude. As he writes, “it is considered wrong to aim at diminishing one’s own consciousness in order to dissociate oneself from one’s troubles, whether great or small. Human beings who aim at diminishing their own consciousness in order to escape their troubles are thought to be diseased, suffering from psychiatric conditions such as drug addiction.”^32^

Let me make two comments regarding the difference between Sulmasy’s argument and mine. Firstly, though different, they may be compatible with each other. To repeat, the difference lies mainly in how broadly or narrowly we construe the bad consequence of sedation. While Sulmasy considers the reduction of consciousness is generally bad, I focus on the badness of total and permanent unconsciousness. Hence, if I accept his broader understanding, I may be led to the same conclusion as his.

Secondly, however, let me also emphasize that the fact that my argument does not employ the broad construal has its merits. For one thing, it is important to see precisely what conclusions will be drawn if we restrict ourselves to the badness of rendering the patient permanently unconscious. More researchers are interested in this narrow construal when the ethics of sedation is discussed. Based on a close examination of the protocol of gradual CDS, my analysis demonstrates that the DDE would not justify most (if not all) cases of PPS that result in CDS, even on the assumption that the badness of sedation should be construed narrowly, i.e., that gradual reduction of consciousness is not morally problematic in itself.

Additionally, there is also a reason to avoid the broad construal. Sulmasy’s claim that reduction of consciousness in itself always deserves strong moral disapproval seems controversial. There are cases where a terminally ill patient accepts mild reduction of consciousness to avoid serious suffering. The claim sounds less plausible as we consider very mild cases.^33^

## 5 CONCLUSIONS

That it has the effect of making the patient permanently unconscious is clearly an undesirable aspect of CDS. In this paper I have argued against the claim that physicians who practice CDS do not intend permanent unconsciousness if they begin with a small amount of sedatives and increase them gradually. If my arguments are valid, the DDE would not be useful in justifying most cases of CDS. Further, because it is plausible to assume that being rendered permanently unconscious is just as bad as being dead, and because CDS induces permanent unconsciousness just as surely as euthanasia causes death, it would be difficult to expect that DDE establishes a moral difference between CDS and euthanasia.

## CONFLICT OF INTEREST

The author declares no conflict of interest.

## Footnotes

^1^ Rys, S., Deschepper, R., Mortier, F., Deliens, L., Atkinson, D., & Bilsen, J. (2012). The moral difference or equivalence between continuous sedation until death and physician‐assisted death: Word games or war games? *Bioethical Inquiry, 9*, 171–183. According to Rys and colleagues, 37 papers that compared the morality of continuous sedation and physician‐assisted suicide were published in the medical and nursing journals from 1966 to 2009. Among them, only two papers raised the point that sedation reduces or removes consciousness. Most authors focused on other questions such as whether sedation shortens life, whether physicians who practice sedation intend to shorten life, and whether the amount of sedatives used is always proportionate to the severity of symptoms.

^2^ See Maeda, I., Morita, T., Yamaguchi, T., Inoue, S., Ikenaga, M., Matsumoto, Y., … Kinoshita, H. (2016). Effects of continuous deep sedation on survival in patients with advanced cancer. *Lancet Oncology, 17*, 115–122.

^3^ Twycross, R. (2019). Reflections on palliative sedation. *Palliative Care: Research and Treatment, 12*, 1–16. Although it is sometimes disputed, this paper assumes that sedation can induce complete unconsciousness.

^4^ Matersvedt, L.J., & Bosshard, G. (2009). Deep and continuous palliative sedation (terminal sedation): Clinical‐ethical and philosophical aspects. *Lancet Oncology, 10*, 622–627; Juth, N., Lindblad, A., Lynoe, N., Sjostrand, M., & Helgesson, G. (2013). Moral differences in deep continuous palliative sedation and euthanasia. *BMJ Supportive & Palliative Care, 3*, 203–206.

^5^ Strictly speaking, DDE is not needed for this line of defense of CDS. What is necessary is a less loaded idea that when two instances of actions have equally bad consequences, and one is done with the intention to bring about this consequence while the other is not, then the latter is less objectionable than the former. While DDE is usually employed to ground the idea, there are other ways to do this as well. For example, a Kantian principle that one ought not to intend to treat a rational being merely as a means to promote or maintain people’s well‐being should also assure its truth in some cases. (The principle may deem CDS to be impermissible if the practicing physician intends to make the patient permanently unconscious because doing so amounts to treating a person’s existence as something of value merely as a means to sustain a tolerable quality of life, i.e., only insofar as the person has a tolerable quality of life, but permissible if the physician does not have this intention.) My criticism applies to any argument that defends CDS based on this more general idea.

^6^ For example, a survey of Dutch physicians showed that 14% of the respondents had a partial intention to hasten death when sedating patients: Swart, S. J., Brinkkemper, T., Rietjens, J. A. C., Blanker, M. H., Zuylen, L., Ribbe, M., … Perez, R. S. G .M. (2010). Physicians’ and nurses’ experiences with continuous palliative sedation in the Netherlands. *Archives of Internal Medicine, 70*,(14), 1271–1274.

^7^ Matersvedt & Bosshard, *op. cit*. note 4; Juth et al. *op. cit*. note 4; LiPuma, S.H. (2013). Continuous sedation until death as physician‐assisted suicide/euthanasia: A conceptual analysis. *Journal of Medicine and Philosophy, 38*, 190–204; Sulmasy, D. P. (2018). The last low whispers of our dead: When is it ethically justifiable to render a patient unconscious until death? *Theoretical Medicine and Bioethics, 39*, 233–263.

^8^ That physicians have this intention is simply assumed in the arguments of the following articles: Juth et al. op. cit. note 4; LiPuma, op. cit. note 7.

^9^ Quill, T. E., Lo, B., Brock, D. W., & Meisel, A. (2009). Last‐resort options for palliative sedation. *Annals of Internal Medicine, 151*, 421–424.

^10^ Cellarius, V., & Henry, B. (2010). Justifying different levels of palliative sedation. *Annals of Internal Medicine, 152*, 332; Sulmasy, D. P., Brungardt, G. S., & Gavanaugh, T. (2010). Justifying different levels of palliative sedation. *Annals of Internal Medicine, 152*, 332–333; Putman, M. S., Yoon, J. D., Rasinki, K. A., & Curlin, F. A. (2013). Intentional sedation to unconsciousness at the end of life: Findings from a national physician survey. *Journal of Pain and Symptom Management, 46*(3), 326–334; Morita, T., Imai, K., Yokomichi, N., Mori, M., Kizawa, Y., & Tsuneto, S. (2017). Continuous deep sedation: A proposal for performing more rigorous empirical research. *Journal of Pain and Symptom Management, 53*(1), 146–152; Hamano, J., Morita, T., Ikenaga, M., Abo, H., Kizawa, Y., & Tunetou, S. (2018). A nationwide survey about palliative sedation involving Japanese palliative care specialists: Intentions and key factors used to determine sedation as proportionally appropriate. *Journal of Pain and Symptom Management, 55*(3), 785–791. For discussions, see also Twycross, op. cit. note 3; Sulmasy, op cit. note 7.

^11^ The distinction was first suggested by Quill, et al. op. cit. note 9. The terms “gradual CDS” and “rapid CDS” were introduced by Morita et al. op. cit. note 10. Morita and colleagues recommend that the distinction is defined solely by the difference in protocols when it is employed in empirical studies because the difference in intentions is difficult to identify by empirical measures. My descriptions of the two types basically follow Quill and colleagues’, but I also explain the deviations in my text below.

^12^ Seymour, J., Rietjens, J., Bruinsma, S., Deliens, L., Sterckx, S., Mortier, F., … van der Heide, A. (2015). Using continuous sedation until death for cancer patients: A qualitative interview study of physicians’ and nurses’ practice in three European countries. *Palliative Medicine, 29*(1), 48–59.

^13^ Ibid. There is an apparent equivocation in these descriptions. The descriptions (and also the name “PPS”) appear to assume that a rapid increase of the medication is never proportionate to the severity of symptoms, but this may not be true. In cases where the physician administers a heavy dose right from the start, believing that it is the minimum amount necessary to control the symptoms, it is not clear from the descriptions whether the practice is considered gradual or rapid.

^14^ Quill et al. op. cit. note 9, pp. 421–422.

^15^ Ibid: 423.

^16^ See articles in note 10.

^17^ Quill et al. op cit. note 9, p. 421.

^18^ Morita et al. op. cit. note 10, p. 148.

^19^ The two terms are understood as synonymous in Twycross op. cit. note 3.

^20^ Quill et al. op. cit. note 9, p. 442.

^21^ Cellarius & Henry, op. cit. note 10, p. 332.

^22^ Sulmasy, D. P. (2007). “Reinventing” the rule of double effect. In B. Steinbock, *The Oxford Handbook of Bioethics* (pp. 114–149). Oxford: Oxford University Press, p.134.

^23^ Ibid: 136.

^24^ Ibid: 140.

^25^ Ibid: 133. However, Sulmasy also gives some rationale behind the condition (p. 123).

^26^ These points and examples I owe to David Wendler.

^27^ The distinction between PPS and PSU recently motivated some researchers to investigate physicians’ intentions empirically. In a questionnaire survey conducted in the USA in 2010, physicians were asked if they intended permanent unconsciousness when sedating patients, and only 10% of the participants responded positively. The authors concluded that “intentionally sedating dying patients to unconsciousness until death is neither the norm in clinical practice nor broadly supported” (Putnam et al. op. cit. note 10, p. 326). The problem of this survey is that it fails to provide the evidence that the physicians’ self‐reports about their intentions are not implausible. The same comment applies to a similar survey conducted in Japan in 2016 (Hamano et al. op. cit. note 10).

^28^ Sulmasy, op. cit. note 7, p. 243.

^29^ Ibid: 248.

^30^ Ibid: 250.

^31^ This is the way Sulmasy must be understood to explain how DDE prohibits PPS in the new article. However, I do not think Sulmasy seriously believes that one can both act to diminish someone’s consciousness as a means to stop suffering and plausibly deny the intention to do so, because the belief apparently contradicts the plausibility condition he proposed in the earlier article.

^32^ Sulmasy, op. cit. note 7: pp. 238–239.

^33^ Sulmasy (op. cit. note 7) in fact provides two exceptions to his opposition to the use of palliative sedation. One is when the symptoms are so severe that the patient cannot experience “the goods of consciousness,” such as “love, prayer, moral choice, speculative thought, aesthetic experience, and so on” (p. 253). The other is the case of intermittent deep sedation (p. 257). However, for Sulmasy, these exceptions are justified not by DDE but by other reasons (which I shall not recount here). Also, the mild cases of consciousness‐reduction are not included in the exceptions.

